# Anchor-Free Localization Method for Mobile Targets in Coal Mine Wireless Sensor Networks

**DOI:** 10.3390/s90402836

**Published:** 2009-04-21

**Authors:** Zhongmin Pei, Zhidong Deng, Shuo Xu, Xiao Xu

**Affiliations:** 1 State Key Laboratory of Intelligent Technology and Systems, Tsinghua National Laboratory for Information Science and Technology, Department of Computer Science and Technology, Tsinghua University, Beijing 100084, P.R. China; E-Mail: x-xu07@mails.tsinghua.edu.cn (X.X.); 2 Institute of Scientific and Technical Information of China, Beijing 100038, P.R. China; E-Mail: xush@istic.ac.cn (S.X.)

**Keywords:** Wireless sensor networks, Multi-dimensional scaling, Received signal strength, Rank sequence

## Abstract

Severe natural conditions and complex terrain make it difficult to apply precise localization in underground mines. In this paper, an anchor-free localization method for mobile targets is proposed based on non-metric multi-dimensional scaling (Multi-dimensional Scaling: MDS) and rank sequence. Firstly, a coal mine wireless sensor network is constructed in underground mines based on the ZigBee technology. Then a non-metric MDS algorithm is imported to estimate the reference nodes’ location. Finally, an improved sequence-based localization algorithm is presented to complete precise localization for mobile targets. The proposed method is tested through simulations with 100 nodes, outdoor experiments with 15 ZigBee physical nodes, and the experiments in the mine gas explosion laboratory with 12 ZigBee nodes. Experimental results show that our method has better localization accuracy and is more robust in underground mines.

## 1. Introduction

Over the past decade, there has been a surge of accidents in coal mines all over the world. Realization of environment monitoring and miner localization in underground mines plays an important role in mining safety. Wireless sensor networks (Wireless Sensor Networks: WSN) have attracted more and more research interest in coal mine applications for their advantages of self-organization, low cost and high reliability. Supported by the British Department of Trade and Industry, the Exeter College Camborne Mining Institution has constructed a high reliable wireless mesh network in mines [1]. Ohio State University has also carried out a WSN project for miner positioning and tracking in the U.S. [2]. Carnegie Mellon University has established a real-time coal mine WSN platform: FireFly [3]. Xia *et al.* have studied WSN design for mobile control applications [4].

Localization algorithms in WSN can be divided into two classes: anchor-based algorithms and anchor-free algorithms [5]. Anchor-based algorithms assume that all reference nodes are anchor nodes or nodes whose real position coordinates are known in advance. Anchor-free localization algorithms only require a few anchor nodes. The coordinates of all the reference nodes are estimated automatically. Typical anchor-free localization algorithms proceed as follows:

1. Estimate the coordinates of the reference nodes. Several methods for this process have been proposed. Meerens and Fitzpatrick use one-hop neighbors and multilateration to construct a global coordinate system [6]. Shang and Ruml use multi-dimensional scaling (Multi-dimensional Scaling: MDS) to realize localization, which has drawn much attention recently [7].
2. Complete precise localization for mobile targets based on reference nodes. Oh-Heum *et al.* present a map stitching localization method in large scale WSN [8]. Kiran and Bhaskar put forward a sequence-based localization (Sequence-based Localization: SBL) method [9].

The above algorithms have respectively achieved certain goals under ideal environments. However, in underground mines, localization will face the following challenges.

1. Water-vapor and coal dust will potentially absorb the wireless signal in different ways and lead to large localization errors.
2. The complex terrain and irregular network topology in underground mines make many localization algorithms do not work well.

To solve the above problems, an anchor-free localization method in coal mine WSN (Coal Mine Wireless Sensor Networks: C-WSN) is proposed. The main contributions of this paper are as follows:

1. A coal mine wireless sensor network is constructed in underground mines based on the ZigBee technology.
2. Non-metric MDS algorithm is introduced into the estimation of the reference nodes’ location, which provides higher fault-tolerance ability.
3. An improved SBL algorithm, *N*-best SBL, is proposed to improve the localization accuracy.

The remainder of the paper is organized as follows. In Section 2, we describe the MDS and SBL method briefly. In Section 3, our anchor-free localization method in C-WSN is studied. In Section 4, we analyze our experimental results. Finally, we conclude the paper.

## 2. Preliminaries

### 2.1. Non-metric MDS algorithms

MDS algorithms are widely used in multivariate statistics. There are two types of MDS algorithms: metric MDS and non-metric MDS. The input in the metric MDS approach is a rigid distance matrix that specifies distances between every pair of nodes, and the output is a coordinate set of all the nodes. The metric MDS approach has been introduced into WSN localization in previous work [7,11]. Compared to the metric MDS approach, non-metric MDS only requires the monotonicity of a similar relationship matrix. In this paper, we take the RSS (Received Signal Strength: RSS) matrix as the input to non-metric MDS and define the RSS matrix as *W*. The RSS can be measured between two adjacent nodes. If some pairs of nodes are not adjacent, we use the shortest path algorithm to estimate the RSS between them.

Without loss of generality, let’s assume that *n* nodes in C-WSN are deployed in *p* dimension space, then the relative coordinate and absolute coordinate of any node *i*(*i* = 1, 2, …, *n*) can be denoted as *R_i_* = (*R*_*i*, 1_, *R*_*i*, 2_, …, *R_i, p_*) and *A_i_* = (*A*_*i*, 1_, *A*_*i*, 2_, …, *A_i, p_*) respectively. Here we focus on the case *p* = 2. Steps of the non-metric MDS algorithm are given as follows.

Step 1: Initialize the node’s coordinate *R_i_* and the number of iterations *k*:
  $$R_{i}=\left(R_{i,1}^{0},R_{i,2}^{0},\cdots ,R_{i,p}^{0}\right)(i=1,2,\cdots n)\;,k\leftarrow 0$$
Step 2: For all node pairs, compute their Euclidean distances:
  (1) $$d_{i,j}^{k}\leftarrow \sqrt{\sum_{t=1}^{p}{\left(R_{i,t}^{k}-R_{j,t}^{k}\right)}^{2}}$$
Step 3: For ${\left(d_{i,j}^{k}\right)}_{n\times n}$ and RSS matrix *W*, calculate the matrix ${\left({\hat{d}}_{i,j}^{k}\right)}_{n\times n}$ using step-wise monotone regression by Equation 2 and Equation 3, i.e. for ∀*i*, *j*, *u*, *v*,
  (2) $${\hat{d}}_{i,j}^{k}\leftarrow \{\begin{array}{ll} \left(d_{i,j}^{k}+d_{u,v}^{k}\right)/2, & \text{if}\;w_{i,j}<w_{u,v}\;\text{while}\;d_{i,j}^{k}>d_{u,v}^{k}\\ d_{i,j}^{k}, & \text{if}\;w_{i,j}<w_{u,v}\;\text{and}\;d_{i,j}^{k}\leq d_{u,v}^{k} \end{array}$$
  (3) $${\hat{d}}_{u,v}^{k}\leftarrow \{\begin{array}{ll} \left(d_{i,j}^{k}+d_{u,v}^{k}\right)/2, & \text{if}\;w_{i,j}<w_{u,v}\;\text{while}\;d_{i,j}^{k}>d_{u,v}^{k}\\ d_{u,v}^{k}, & \text{if}\;w_{i,j}<w_{u,v}\;\text{and}\;d_{i,j}^{k}\leq d_{u,v}^{k} \end{array}$$
Step 4: Compute the stress defined by the Equation (4). If stress < *ε* (here *ε =* 10^−4^), then finish; Otherwise continue to Step 5.
  (4) $$\text{stress}=\sqrt{\frac{\sum_{i=1}^{n-1}\sum_{j=i+1}^{n}{\left(d_{i,j}^{2}-{\hat{d}}_{i,j}^{2}\right)}^{2}}{\sum_{i=1}^{n-1}\sum_{j=i+1}^{n}d_{i,j}^{4}}}$$
Step 5: Update *k* ← *k* + 1, and compute the new node coordinates $\left(R_{i,1}^{k},R_{i,2}^{k},\cdots ,R_{i,p}^{k}\right)$ as follows:
  (5) $$R_{i,t}^{k}=R_{i,t}^{k-1}+\frac{\alpha }{n-1}\sum_{\begin{array}{l} j=1\\ j\neq i \end{array}}^{n}\left(1-\frac{{\hat{d}}_{i,j}^{k-1}}{d_{i,j}^{k-1}}\right)\left(R_{j,t}^{k-1}-R_{i,t}^{k-1}\right),t=1,2,\cdots ,p$$
  where *α* is the iterative step. Then return to Step 2.

As the iterative algorithm grows, the stress will decrease monotonically. It can be shown that *R* will converge to a stationary point [13]. It is worth mentioning that Equations 2 and 3 are to ensure that if *w_i,j_* < *w_u,v_*, then *d̂_i,j_* ≤ *d̂_i,j_*, which is a typical requirement by non-metric MDS.

### 2.2. Sequence-based localization

**Figure 1.**
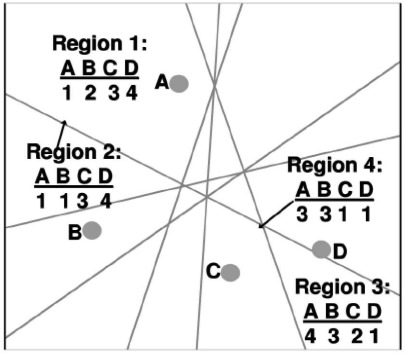
Example of rank sequences for four anchor nodes.

The sequence-based localization method is a novel and high-accuracy anchor-based WSN localization technique, which was recently proposed by Kiran and Bhaskar [9]. The 2D localization space is divided into distinct regions by the perpendicular bisectors of lines joining pairs of anchor nodes. Each region is uniquely identified by a rank sequence that represents the distance ranks of anchor nodes to that region. Figure 1 is an example of rank sequences for four anchor nodes [9].

The process to calculate the localization of mobile targets based on SBL is as follows [9]:

1. Determine all feasible location sequences in the localization space and store them in a location sequence table.
2. Obtain the location sequence of the mobile node by measuring RSS.
3. Search the location sequence table for the “nearest” sequence to the location sequence of the mobile node.
4. Take the centroid of the region, which is presented by the “nearest” location sequence, as the position of the mobile node.

**Figure 2.**
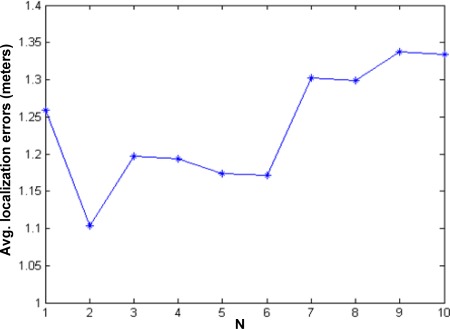
Localization errors due to *N.*

However, based on our detailed observation, we find that it is not optimal in terms of average localization errors if only one “nearest” sequence is searched in the sequence table. Figure 2 shows our experimental results with original outdoor MICA2 data in paper [9]. We select the top *N* “nearest” sequences instead of one sequence when searching the location sequence table. In this case, when *N* = 2, the minimal average localization error can be obtained.

**Figure 3.**
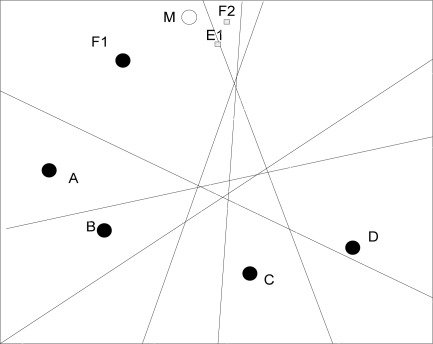
Localization for marginal nodes.

In addition, we also notice that the localization errors for nodes near the border of the region are possibly rather large. For example, in Figure 3, when mobile node *M* falls into region *F1*, its coordinate will be estimated as the centroid of *F1* if no measurement errors exist. In fact, the real position of *M* is closer to the centroid of region *E1*, even *F2*.

To reduce the average localization errors and improve the localization accuracy for marginal nodes, a new sequence-based localization method: *N*-best SBL, is presented. The best *N* is first estimated by using random sampling based on a wireless channel fading model, and then the coordinate of the mobile target is obtained with weighted computing of top *N* sequences.

## 3. Anchor-Free Localization Method in C-WSN

### 3.1. Coal mine wireless sensor networks

**Figure 4.**
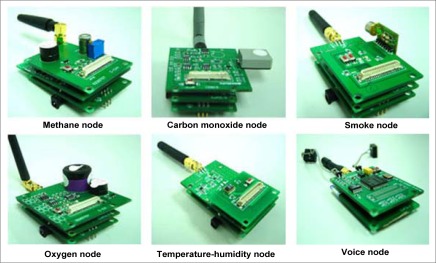
Circuit boards of Cicada sensor nodes.

**Figure 5.**
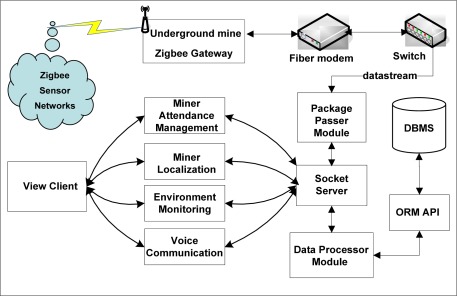
Distributed system architecture for C-WSN.

**Figure 6.**
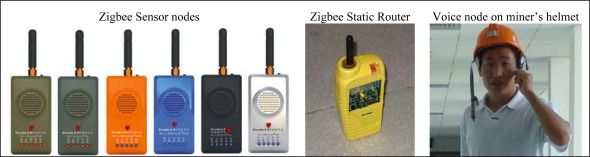
Pictures of Cicada physical nodes.

To execute our localization algorithm, first a C-WSN was constructed in underground mines based on the ZigBee technology. We deployed the sensor nodes, called Cicada, as end devices in the C-WSN. There are six types of nodes including methane sensors, oxygen sensors, carbon monoxide sensors, smoke sensors, temperature-humidity sensors and voice sensors, just as shown in Figure 4. These sensor nodes join the C-WSN, acquire the environment information on a fixed time cycle and transmit sensing data to the ZigBee gateway. Static router nodes are previously deployed to construct the ZigBee backbone network. They are also reference nodes for mobile targets. Voice sensor nodes are installed on miner’s helmets. Miners are the mobile targets for localization. The ZigBee gateway collects sensor data and transmits them to the monitoring center. The gateway connects to a fiber modem which can transmit the data transparently. All the information data are processed and displayed in monitoring center with several distributed servers and clients. Four function units are implemented in the C-WSN system: miner attendance management, miner localization, environment monitoring, and voice communication. The distributed system architecture for C-WSN is shown in Figure 5. Figure 6 shows the pictures of Cicada physical nodes.

### 3.2. Anchor-free localization algorithm in C-WSN

Based on the non-metric MDS algorithm and the *N*-best SBL algorithm, an anchor-free localization algorithm in C-WSN is demonstrated in this paper. The localization process is as follows:

1. After C-WSN was established, static ZigBee router nodes start up the non-metric MDS algorithm and then complete the estimation of coordinates with few anchor nodes.
2. With the estimated coordinates of static router nodes, mobile nodes finish the precise localization process by executing the *N*-best SBL algorithm.

The details of the anchor-free localization algorithm are discussed as follows.

#### 3.2.1. Non-metric MDS algorithm for static router nodes

Most of existing WSN localization methods based on the MDS algorithm adopt metric MDS. However, it is hard to obtain precise distance matrix of the nodes in underground mines. Here, non-metric MDS algorithm is used to estimate the coordinates of reference nodes. Under the condition that more than three anchor nodes’ absolute coordinates are known, the reference nodes’ coordinates can be calculated in the following steps:

Step 1: After joining the network, all reference nodes broadcast one-hop RSS request message. The neighbor nodes measure the RSS value between them and report the response message to the sever through the gateway.
Step 2: The sever starts up the Dijkstra's shortest path algorithm to construct the RSS relationship matrix for every pair of nodes, which is the input to the non-metric MDS.
Step 3: Finish the non-metric MDS algorithm process to obtain the relative coordinates of all reference nodes.
Step 4: Compute the absolute coordinates through shifting, translating, rotating and/or reversing with anchor nodes.

The detailed computation process of this step is given as follows:

For convenience, assume the previous *m* (3 ≤ *m* < *n*) nodes are anchor nodes, whose location coordinates are known in advance. By space analytic geometry, all other transformations besides shifting can be performed by the product of the coordinate vector and the transformation matrix. Therefore, one anchor node can be regarded as the origin before transforming. Thus multiple transformations can be completed by multiple products of transformation matrices, i.e.:

(6) $$\begin{array}{l} {\left({\left(A_{2}-A_{1}\right)}^{T},{\left(A_{3}-A_{1}\right)}^{T},\cdots ,{\left(A_{m}-A_{1}\right)}^{T}\right)}^{T}=\\ {\left({\left(R_{2}-R_{1}\right)}^{T},{\left(R_{3}-R_{1}\right)}^{T},\cdots ,{\left(R_{m}-R_{1}\right)}^{T}\right)}^{T}\times Q \end{array}$$

Here *Q* is called the optimal transfer function. To simplify the notations, denote *M*_1_ and *M*_2_ as:

(7) $$M_{1}={\left({\left(A_{2}-A_{1}\right)}^{T},{\left(A_{3}-A_{1}\right)}^{T},\cdots ,{\left(A_{m}-A_{1}\right)}^{T}\right)}^{T}$$

(8) $$M_{2}={\left({\left(R_{2}-R_{1}\right)}^{T},{\left(R_{3}-R_{1}\right)}^{T},\cdots ,{\left(R_{m}-R_{1}\right)}^{T}\right)}^{T}$$

By simple deductions, we have:

(9) $$Q={\left(M_{2}^{T}\times M_{2}\right)}^{-1}\times M_{2}^{T}\times M_{1}$$

If *Q* is known, coordinates of other nodes can be obtained easily by Equation 10:

(10) $$\begin{array}{l} {\left(A_{m+1}^{T},A_{m+2}^{T},\cdots ,A_{n}^{T}\right)}^{T}=\\ {\left({\left(R_{m+1}-R_{1}\right)}^{T},{\left(R_{m+2}-R_{1}\right)}^{T},\cdots ,{\left(R_{n}-R_{1}\right)}^{T}\right)}^{T}\\ \times Q+{\left(A_{1}^{T},A_{1}^{T},\cdots ,A_{1}^{T}\right)}^{T} \end{array}$$

#### 3.2.2. Precise localization for mobile targets based on *N*-best SBL algorithm

After all the reference nodes have obtained their own absolute coordinates based on non-metric MDS, the mobile targets start up the *N*-best SBL algorithm. The localization procedure is as follows:

1. Mobile targets broadcast one-hop RSS request messages at fixed time cycle. After receiving the messages, reference nodes calculate RSS values between them and report them to the server through gateway.
2. The server reads the coordinates information of related reference nodes, starts up *N*-best SBL algorithm, and obtains the position coordinates of mobile node.
3. Return to step (1), repeat the localization process with different reference nodes.

In what follows, the *N*-best SBL algorithm is described in detail. A wireless channel fading model is needed for the *N*-best SBL algorithm. Here, we adopt:

(11) $$P_{R}(d)=P_{T}-\mathrm{PL}(d_{0})-10\eta {\text{log}}_{10}\frac{d}{d_{0}}+X_{{}_{\sigma }}$$

which is widely used in RSS-based WSN localization [10], where *P_R_* is the received signal power, *P_T_* is the transmit power, and *PL* (*d*_0_) is the path loss for a reference distance of *d*_0_. *η* is the path loss exponent, and the random variation in RSS is expressed as a Gaussian random variable of zero mean and *σ*^2^ variance *X_σ_* = *N* (0, *σ*^2^).

Specific procedure of the *N*-best SBL algorithm is as follows:

- Step 1: Estimate the parameters *η* and *σ* in Equation 11 by linear regression and maximum likelihood methods based on the RSS information of reference nodes.
- Step 2: Construct the location sequence table *T* = {*S*_1_, *S*_2_, …, *S*_|*T*|_} from reference nodes.
- Step 3: Estimate the optimal *N* value, denoted as N*.
  Step 3.1: Generate a number of virtual nodes *DN* randomly according to a uniform distribution in the area bounded by *B*.
  Step 3.2: Loop for each *N* in *N* *^val^* = {1, 2, …, 10}.
    Step 3.2.1: Loop for each node (*x*, *y*) ∈ *DN*.
      Step 3.2.1.1: RSS values with reference nodes are simulated by Equation 11, thus a corresponding rank sequence *S* is obtained.
      Step 3.2.1.2: Calculate correlation coefficients, *τ* (*S_i_*), *i* = 1, 2, …, |*T* |, between *S* and each rank sequence *S_i_* in *T* according to Equation 12:
        (12) $$\tau =\frac{\left(n_{c}-n_{d}\right)}{\sqrt{n_{c}+n_{d}+n_{\mathrm{ts}}}\sqrt{n_{c}+n_{d}+n_{\mathrm{tt}}}}$$
        where *n_c_* is the number of concordant pairs, *n_d_* is the number of discordant pairs, *n_ts_* is the number of ties in *S*, and *n_tt_* is the number of ties in *S_i_*.
      Step 3.2.1.3: Sort *T* by correlation coefficients in descending order, and then select top *N* rank sequences from *T*, denoted as *T^N^*.
      Step 3.2.1.4: Estimate the coordinates by Equation 13,
        (13) $$\left(\hat{x},\;\hat{y}\right)=\frac{1}{\sum_{i=1}^{N(T)}\frac{1+\tau (T_{i})}{2}}\sum_{i=1}^{N(T)}\left(\frac{1+\tau (T_{i})}{2}C_{i}\right)$$
        where *N* (*T*) is the number of sequences in *T^N^*, and *C_i_* is the centroid coordinates of the region represented by *S_i_*.
  Step 3.3: Calculate the average location errors for virtual nodes by Equation (14):
    (14) $${\bar{E}}_{N}=\frac{1}{|\mathrm{DN}|\times R}\sum_{(x,y)\in \mathrm{DN}}\left[{\left(x-\hat{x}\right)}^{2}+{\left(y-\hat{y}\right)}^{2}\right]$$
    where *R* is the radius of communication.
  Step 3.4: The optimal *N* value is denoted as follows:
    (15) $$N^{*}=\text{arg}\;\underset{N\in N^{\mathrm{val}}}{\text{min}}\;{\bar{E}}_{N}$$
- Step 4: For any mobile target, measure RSS values with reference nodes and obtain a corresponding rank sequence *S* firstly. Then complete one precise localization process based on Step 3.2.1.2 to Step 3.2.1.4.

The practical significance of the *N*-best SBL location method is that in the area covered by a certain number of reference nodes, the mobile node can obtain the minimum average location errors when moving.

## 4. Experimental Results

The following three steps are used to validate the performance of our algorithm:

1. Firstly, outdoor experiments with 15 real Cicada nodes were carried out to test the performance of the non-metric MDS algorithm..
2. Secondly, 10,000 repeats of simulation experiments with 100 nodes were finished to compare the performance between the *N*-best SBL algorithm and the original SBL algorithm.
3. Finally, the experiments in the mine gas explosion laboratory with our anchor-free localization algorithm were executed to test the whole localization performance.

### 4.1. Outdoor experiments for non-metric MDS

The outdoor experiments were realized in a vacant environment within an area of 60 m × 40 m, where 15 nodes of the Cicada series were randomly distributed. Cicada nodes are designed based on the CC2430 ZigBee chip with a radio frequency power amplifier. The point to point communication distance reaches to 200 m. The experimental process is as follows:

1. Measure the real location coordinates of the 15 nodes after deployment.
2. All the nodes broadcast one-hop RSS request message. The neighbor nodes report the response messages to the server.
3. Construct the RSS relationship matrix for all the nodes in the server.
4. Choose *m* reference nodes (*m* = 3, 4,…14) as anchor nodes randomly, run the non-metric MDS algorithm and estimate the location coordinates for all the nodes according to the RSS relationship matrix.
5. Compute localization errors of the non-metric MDS algorithm according to Equation 14. For each *m*, 15 runs are conducted and then the total average localization errors are obtained.

**Figure 7.**
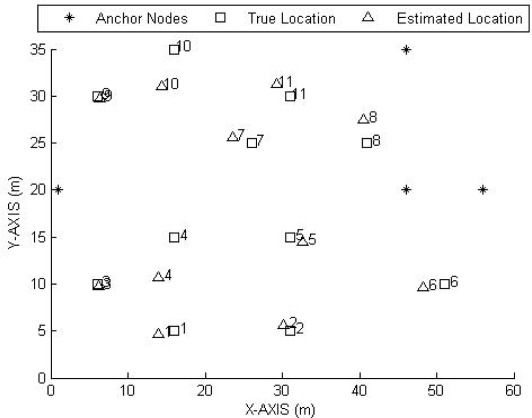
Comparison between true location and estimated location due to non-metric MDS.

Figure 7 illustrates the difference between true locations and estimated locations of reference nodes in outdoor experiments for one run when *m* = 3.

**Figure 8.**
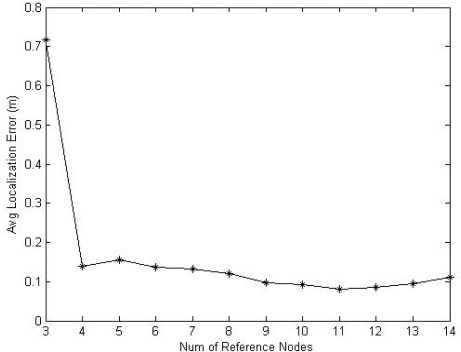
Non-metric MDS performance.

**Figure 9.**
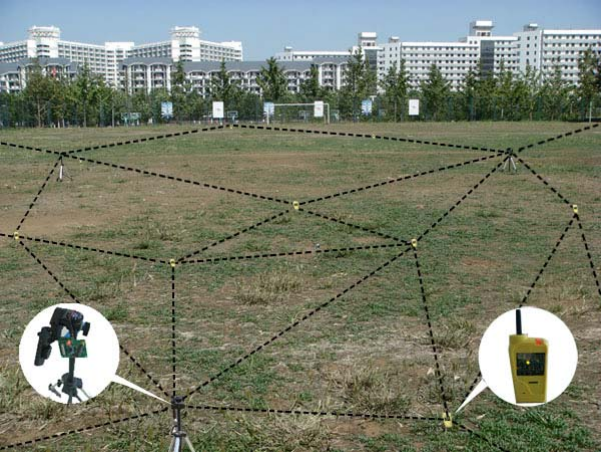
Outdoor experiments.

Figure 8 shows the performance for the non-metric MDS and how the total average localization errors vary with the number *m* of reference nodes. The experimental results in Figure 8 show that the non-metric MDS algorithm has higher localization accuracy, and especially when more than four anchor nodes are available, the algorithm can obtain more effective results. Figure 9 shows the scene of the outdoor experiments.

### 4.2. Simulations for N-best SBL

To verify the performance of the *N*-best SBL algorithm in a large scale WSN, we completed the simulations in MATLAB and present a comparative study with the original SBL algorithm. First, a 100 × 100 square meters localization space is defined, where *n* (*n* = 10) reference nodes are generated randomly and uniformly. The localization space is divided further into 100 grids of the same size, that is, each grid covers 10 × 10 square meters. Then, one virtual localization target node is generated randomly and uniformly in each grid. Thus, there are 100 virtual target nodes in total. Suppose that all nodes are in the radio range of each other. The lognormal shadowing simulation model (Equation 11) is utilized to generate corresponding RSS values. Finally, the localization for each target node is attained with 10 reference nodes, and the average localization errors are calculated, similar to Equation 14. The results in this section are averaged over 100 runs.

**Figure 10.**
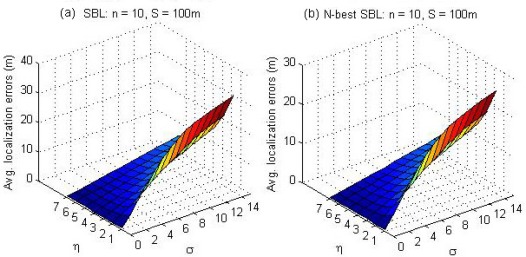
The average localization errors as a function of standard deviation *σ* and path loss exponent *η* for the SBL method (a) and the *N*-best SBL method (b).

Figure 10 illustrates the average localization errors as a function of standard deviation *σ* and path loss exponent *η* for the SBL method (a) and the *N*-best SBL method (b), respectively. From the results reported in Figure 10, it is not difficult to see that the *N*-best SBL method is always superior to the SBL method in all cases. Especially, the superiority is very significant when *η* = 1 and *σ* = 14.

**Figure 11.**
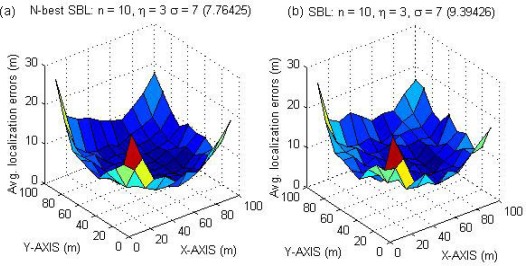
When *η* = 3 and *σ* = 7, the average localization errors in different regions for the *N*-best SBL method (a) and the SBL (b).

Figure 11 shows the average localization errors in different regions for *N*-best SBL (a) and SBL (b) methods, respectively, when *η* = 3 and *σ* = 7 (typical values). The number in parenthesis above each sub-figure is the average localization errors of the whole region. The results in Figure 11 reveal that the average localization errors of the whole region for the *N*-best SBL method are lower than that of the SBL method. What’s more, the minimum localization error of each region for the *N*-best SBL method is always lower than that of the SBL method. However, there exist some regions whose maximum localization error for the *N*-best SBL method is higher than that of the SBL method. On closer examination, we find that the main reason is related to the settings of weights in Equation 13. Some sigmoid functions can be considered in order to raise the contribution of rank sequences whose correlation coefficients are large, and suppress the contribution of others.

**Figure 12.**
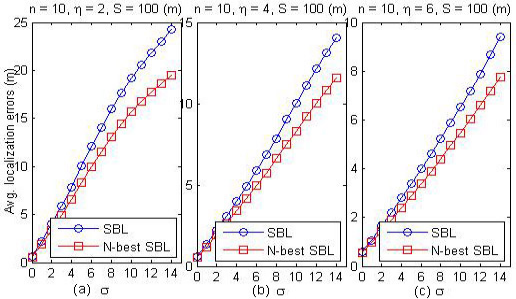
The performance comparison of SBL method and *N*-best SBL method in terms of the average localization errors for *η* = 2 (a), *η* = 4 (b), and *η* = 6 (c).

Figure 12 depicts the performance comparison of the SBL method and the *N*-best SBL method in terms of the average localization errors for *η* = 2 (a), *η* = 4 (b) and *η* = 6 (c), respectively. From the results in Figure 12, we can notice that for the wireless channel fading model by Equation 11, the average localization errors for the *N*-best SBL method are always lower than that of the SBL method when assuming standard deviation *σ* with various values. This indicates that the *N*-best SBL method has higher anti-noise ability.

### 4.3. Experiments in the laboratory of mine gas explosion for anchor-free localization algorithm

**Figure 13.**
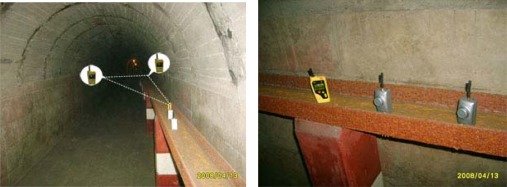
Experiments in the mine gas explosion laboratory.

Since the explosive-proof certification of our mine products was in process, the whole performance study of anchor-free localization method was executed in the mine gas explosion laboratory, which simulates the real environment of underground mines. The average temperature in the laboratory of mine gas explosion is about 24.5 °C and the average relative humidity is about 56.8%. The length of the tunnel is about 160 meters and the width is about 2 meters. Our ZigBee network comprises one gateway node, 10 static router nodes, 16 static sensor nodes and a mobile node. Sensor nodes are not involved in the localization process. Router nodes are deployed in fixed location every 15 meters. They completed location estimation based on the non-metric MDS algorithm with four anchor nodes firstly. Then the mobile node conducted the localization process with the *N*-best SBL algorithm. Figure 13 shows the experimental setup in the laboratory.

**Figure 14.**
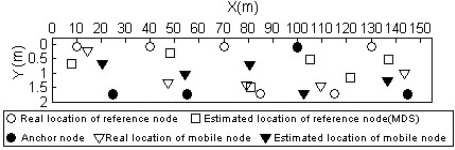
Performance comparison of anchor-free localization method.

Figure 14 shows the performance comparison of the anchor-free localization method. The real location of reference nodes, the estimated location of reference nodes with non-metric MDS algorithm, the real location of the mobile node and the estimated location of the mobile node are illustrated. The anchor-free algorithm was executed in five positions by the mobile node. We calculated the average localization errors according to Equation 14, and got the final result: 0.6936 meters. It is an ideal result for the localization of mobile targets in underground mines.

## 5. Conclusions

An anchor-free localization method for mobile targets is implemented in C-WSN based on non-metric MDS and *N*-best SBL. We constructed a C-WSN for underground mines based on the ZigBee technology and imported the non-metric MDS algorithm into the C-WSN localization. An improved SBL algorithm, *N*-best SBL, is presented to achieve the precise localization for mobile targets. The results of simulation and real-world experiments show that our method has higher localization accuracy. Target tracking is also an important problem for WSN [12]. In our ongoing work, we are studying the tracking method for mobile miners in C-WSN. The experiments for our method in real underground mines will also be carried out.
